# RIDME Spectroscopy: New Topics Beyond the Determination of Electron Spin–Spin Distances

**DOI:** 10.1021/acs.jpclett.4c02667

**Published:** 2025-01-22

**Authors:** Sergei Kuzin, Maxim Yulikov

**Affiliations:** † Department of Chemistry and Applied Biosciences, ETH Zurich, Vladimir Prelog Weg 2, 8093 Zurich, Switzerland

## Abstract

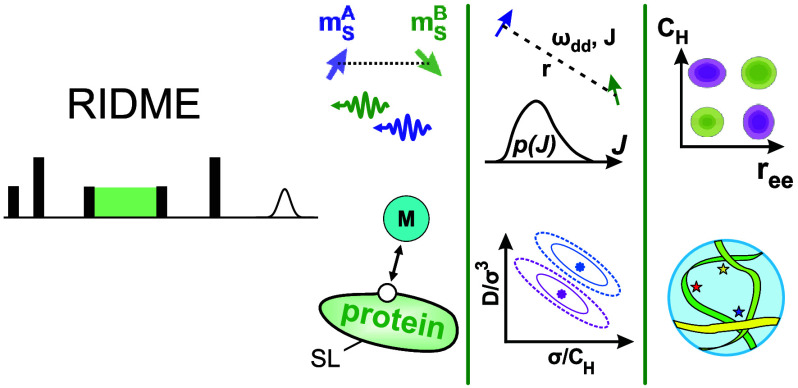

Relaxation-induced dipolar modulation enhancement (RIDME) is a pulse EPR experiment originally designed to determine distances between spin labels. However, RIDME has several features that make it an efficient tool in a number of “nonconventional” applications, away from the original purpose of this pulse experiment. RIDME appears to be an interesting experiment to probe longitudinal electron spin dynamics, e.g., in relation to qubits research, to probe distributions of exchange couplings, useful for the design of molecular magnets, and to determine important details of electron spin interactions with the nuclear spin bath, which is related to the dynamic nuclear polarization and soft materials research. We also anticipate interesting applications of RIDME in the structural biology of biopolymers as well as their interactions, aggregation, and phase separation. It is not excluded that in the near future such “nonconventional” topics could grow in number and evolve into the main application area of RIDME.

Relaxation-induced dipolar modulation enhancement (RIDME) is mostly known as one of the pulse dipolar EPR spectroscopy (PDS) experiments,^1,2^ which gained attention particularly in structural biology applications as a method to determine site-to-site distance constraints based on magnetic dipolar couplings between site-selectively introduced spin labels.^3,4^ In this context, however, the well-known double electron–electron resonance (DEER) experiment^5−9^ is considered the primary PDS technique to determine the intramolecular electron–electron magnetic dipolar couplings and related distance constraints.^10−13^ The RIDME experiment proved to be useful for fast-relaxing paramagnetic species, in particular for paramagnetic-metal-based spin labels,^14−18^ as well as for PDS measurements at ambient temperature.^19^ Nevertheless, to date, its applications as a PDS technique are limited compared to DEER, even though the basic methodology for RIDME data acquisition and processing has been developed.^20−23^ Partially, this is related to a more complex composition of the RIDME signal, which depends on the spin label relaxation properties^20,21^ and includes, apart from the electron dipolar couplings, also strong contributions due to the electron–nuclear interactions.^21,24^

In our view, precisely these features of the RIDME experiment, which narrow down its use in PDS, open up roads toward interesting applications where RIDME stands out as a far more efficient pulse EPR experiment to exploit. A graphical overview of such applications, presented in Figure 1, is grouped into six blocks, labeled I–VI. The direct relation of the RIDME signal to the relaxation of the partner spin allows us to study the stochastic spin-flip dynamics in coupled spin pairs (Figure 1, I). Such spin pair dynamics might have some interesting features overlooked in earlier studies. In particular, analyzing RIDME data will reveal details on probabilities and time scales of simultaneous flip–flips or flip–flops in pairs of coupled spins. Next, the substitution of the pump pulse, which is always bandwidth-limited, by a relaxation-driven evolution block turns RIDME into an essentially infinite-pump-bandwidth technique. This feature can be utilized in some important applications, such as measurements of metal ion affinity to protein binding sites (Figure 1, II)^25−28^ and measurements of exchange couplings in weakly coupled metal-based molecular-magnet-like systems (Figure 1, III).^29^

**Figure 1:**
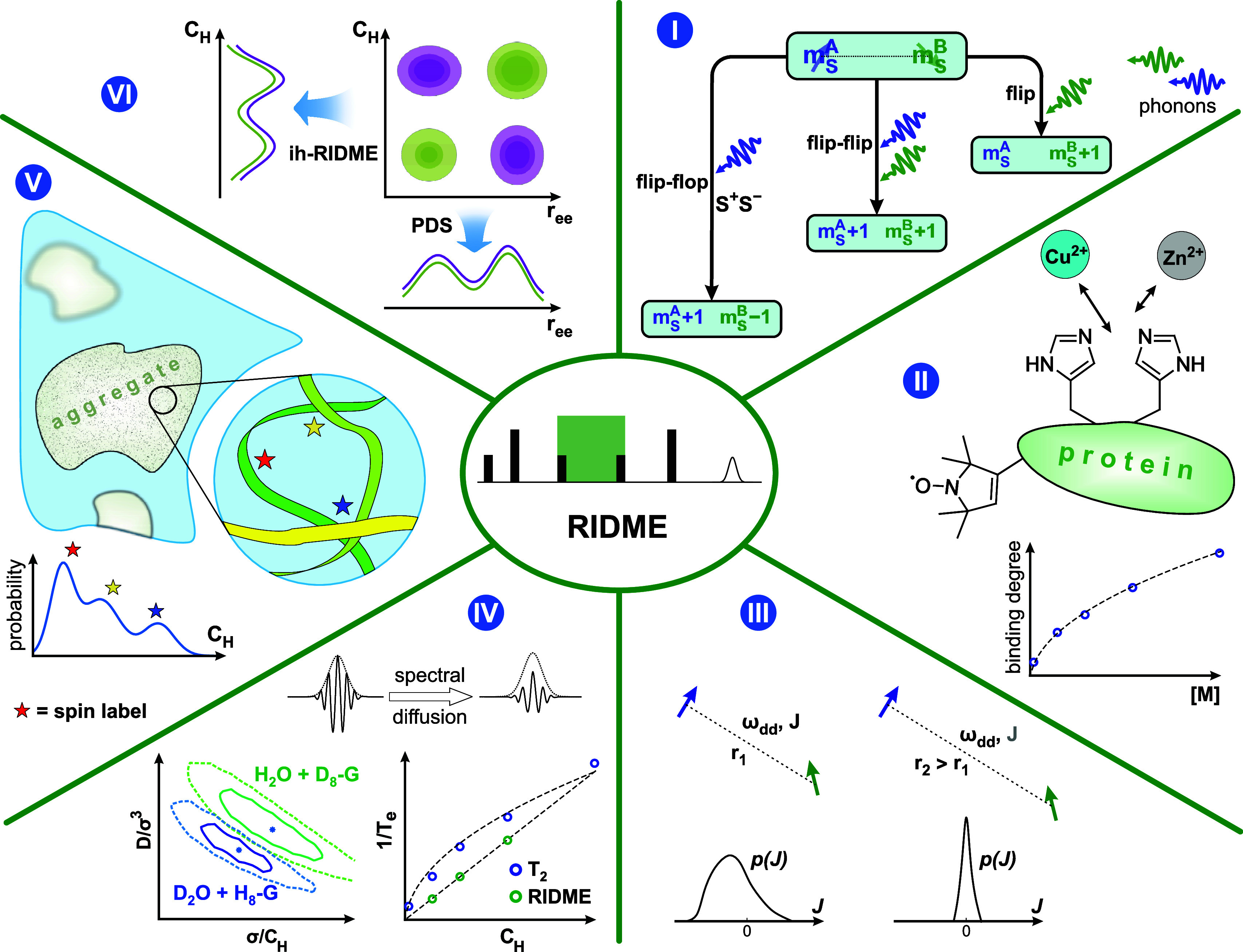
Overview of the applications of the RIDME experiment. The RIDME pulse sequence is presented in the middle round panel, with π and π/2 pulses differentiated by height. The mixing block consists of two π/2 pulses separated by a delay time, *T*_mix_ (highlighted in green). RIDME experiments can be involved in (I) investigation of spin-flip probabilities and spin–phonon interactions in systems of coupled electron spins; (II) metal binding affinity measurements in vitro and in cells; (III) studies of intramolecular Heisenberg exchange couplings and their distributions; (IV) quantification of electron-spin decoherence rates due to electron–nuclear and nuclear–nuclear interactions; (V) investigation of weak interactions of macromolecules; and (VI) finding and quantifying the correlations between local proton distributions and electron spin–spin distances. The Roman numerals refer to the corresponding sections in the main text. The plots in panel (IV) were adapted from ref (24). CC BY-NC 3.0.

Another set of important applications of RIDME comes from its ability to probe electron–nuclear interactions beyond the spin diffusion barrier for a rather broad range (up to 3 nm) of electron–nuclear distances around the paramagnetic center without a direct radiofrequency (RF) excitation.^24^ This results in the possibility of measuring the concentration of proton spins near paramagnetic centers in homogeneous samples (Figure 1, IV), the possibility of quantifying the distribution of such local proton concentrations in heterogeneous samples (Figure 1, V), and the potential possibility to correlate the local proton concentration and electron spin–spin distance in heterogeneous samples (Figure 1, VI). Spectroscopic properties of the nuclear spin bath (most importantly, the proton bath) and its time evolution and interaction with electron spin centers are among the primary objects of attention in dynamic nuclear polarization (DNP),^30,31^ and they can be accessed by RIDME. Detecting and quantifying the distribution of vicinal protons can also report on the local heterogeneity in soft materials in cases where one can introduce spin probes. In structural biology, the use of deuterated buffers and protonated spin-labeled biomolecules also induces a “proton concentration heterogeneity”, which can be quantified by RIDME and used to obtain important characteristics of the biomolecules’ structural ensembles, for instance, the statistics of intermolecular contacts.^32,33^ Characterization of conformational ensembles of unstructured biomolecules, such as intrinsically disordered proteins (IDPs) or protein regions (IDRs) or single-stranded RNAs (ssRNAs),^34−36^ as well as other natural polymers (such as polysaccharide soluble dietary fibers)^32,37^ attracts growing attention. Here, the RIDME data might enrich the accessible information, thus complementing other techniques like small-angle scattering,^38^ FRET,^39^ and DEER.^11,40^

To summarize, in this Perspective we overview a handful of promising options for employing the RIDME technique in different currently active research areas beyond the standard PDS application of determining spin–spin distances and distance distributions. Some of these options were suggested recently and are currently in the process of being established as robust spectroscopic tools that might be particularly attractive to pulse EPR methodology-oriented researchers.

## Brief Overview of the RIDME Experiment

**Figure 2:**
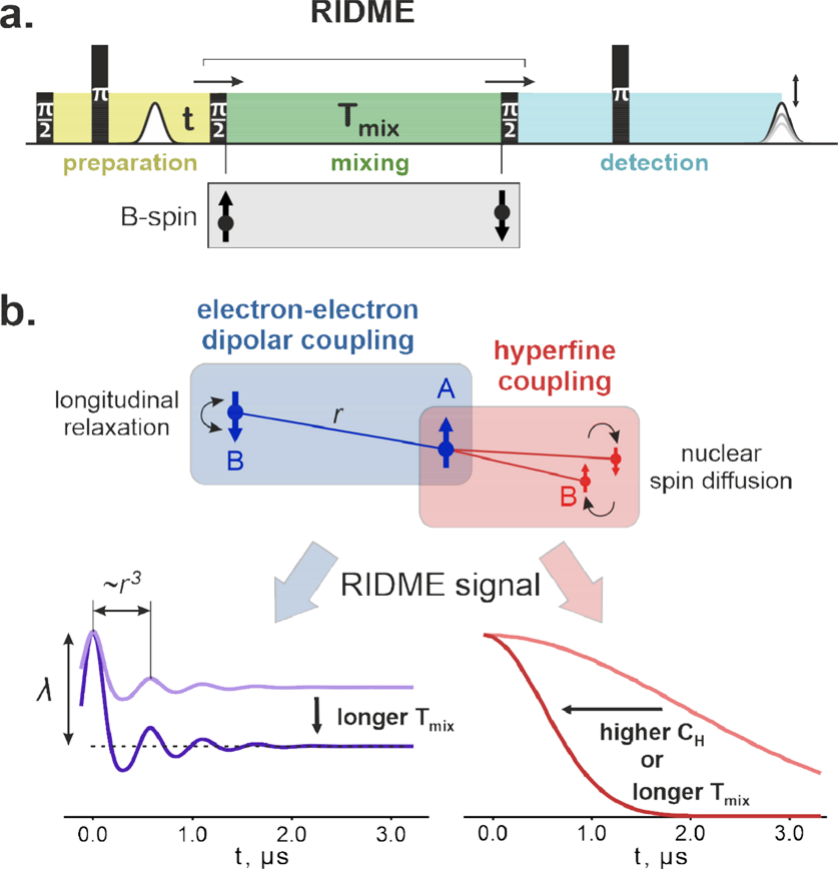
Overview of the RIDME pulse EPR experiment. (a) RIDME pulse sequence. The pulse labels correspond to their flip angles. The colors highlight the preparation (yellow), mixing (green), and detection blocks of the experiment. During the mixing block of length *T*_mix_, the partner spins (electron or nuclear spin, denoted as “B-spin”) change their state. Due to this, the shift of the mixing block position causes the modulation of the echo intensity, which is recorded as a RIDME trace. (b) Two main sources of signal generation in the RIDME experiment: electron–electron dipolar coupling and hyperfine coupling. The longitudinal relaxation of an electron results in a characteristic modulation pattern that directly encodes the interspin distance (*r*) if the Heisenberg exchange interaction is negligible. The relative intensity of the modulation is called the modulation depth (λ). Setting a longer mixing time leads to a larger modulation depth due to a larger probability of the B-spin flip. The evolution of the nuclear states is driven by nuclear spin diffusion. The corresponding modulation effect is stronger at higher nuclear concentrations or longer mixing times. All traces in this figure were generated numerically.

The pulse scheme of the most commonly used five-pulse RIDME experiment is shown in Figure 2a. The primary echo (generated from the first pulse (π/2) and second pulse (π) in the sequence) is refocused by applying another π pulse (the fifth pulse in the sequence). This is the observer sequence part, essentially the refocused Hahn echo sequence, which is the same as in the four-pulse DEER experiment.^5^ The key feature of the RIDME experiment is the mixing block formed by the third and fourth pulses (both π/2) in the sequence. With respect to the mixing block, the preceding and following parts of the sequence can be referred to as the preparation and detection blocks, respectively. During the free evolution period *t* after the primary echo, the electron spin coherence obtains the phase Ω_prep_*t*, where Ω_prep_ is the local magnetic field (in frequency units) experienced by a spin packet during the preparation block. As a result, the ensemble magnetization is modulated with cos Ω_prep_*t*.

The first pulse of the mixing block drives half of the electron coherence in the transverse plane to a longitudinal nonequilibrium polarization grating. The longer the defocusing evolution period the magnetization experienced before the mixing block, the finer is the grating resolution on the frequency scale. Note that the residual half of the electron spin coherence, which was not transferred to the *z* direction, irreversibly dephases during the mixing time and may be disregarded. The second pulse returns the magnetization grating to the transverse plane (i.e., reconverts polarization into coherence), which allows, after free evolution during the detection part, to obtain a spin echo. If the spin environment of the manipulated electron spin does not change in the mixing block, the local magnetic field is preserved, and the spin ensemble perfectly refocuses at any position of the mixing block.

To facilitate further discussion, the manipulated electron spin is called “detected spin” or simply “A-spin”. The other spins in the neighborhood, electron or nuclei, are then called “relaxing spins” or B-spins. These spins are normally not affected directly by the microwave pulses and can be in their eigenstates, and their role consists of forming the local magnetic offset to the detected spin’s resonance field in the form of the electron–electron dipolar coupling or hyperfine couplings.

Storing the magnetization along the external magnetic field allows for tunable mixing times in the RIDME experiment, in particular for long mixing times, substantially beyond the typical transverse relaxation limited durations. Such long waiting times are sufficient to detect the spontaneous change of the B-spin’s magnetic state. When this happens, the local magnetic field in the detection block (Ω_det_) is not equal to Ω_prep_, and upon shifting of the mixing block, the echo intensity is modulated with cos (Ω_prep_ – Ω_det_)*t*. For the electrons, the state flip is governed by incoherent longitudinal relaxation mechanisms (Figure 2b). The frequency of the resulting electron spin echo signal modulation is determined by the distance *r* to the relaxing electron’s spin. The value of the modulation effect, called the modulation depth (λ), is an immediate descriptor of the B-spin’s flip probability. Thus, in the PDS version of the RIDME experiment, the mixing times are set to be comparable to the longitudinal relaxation times of the B-spins to secure substantial electron spin flip probabilities. The idealized buildup kinetics of the modulation depth in RIDME is described by
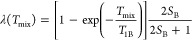
where *S*_B_ is the spin of the relaxing center and *T*_1B_ is its longitudinal relaxation time. We suggest later in this work that following the actual dependence of the modulation depth on the mixing time in RIDME is an informative way to study the spin–lattice relaxation kinetics in high-spin centers or in coupled electron systems complementing, e.g., the inversion recovery measurements (see section I).

Another case of B-spin fluctuation is the nuclear spin diffusion effect that causes the variation of the hyperfine interaction (Figure 2b). The nuclear magnetic moments are substantially smaller than those of electrons (at least ∼660 times for protons). Consequently, the oscillation period of the signal modulation is much greater than several microseconds, which is a typical length of RIDME traces. Eventually, the slow modulations from multiple nuclear pairs surrounding the electron overlay and form a smooth signal decay due to destructive interference. We refer to this contribution as nuclear background. Its steepness and shape are connected to the nuclear density and are also dependent on the length of the mixing block. Here, besides the option of mixing time control, RIDME has another advantage compared to simpler decoherence measurements, such as Hahn echo decay and Carr–Purcell experiments: the RIDME decay series for paramagnetic centers in a proton bath can be rather accurately described by a spectral diffusion model with only a few fitting parameters.^24^ Thus, nuclear spin diffusion in the solid state in the vicinity of a paramagnetic site, which is considered a slow process on the EPR time scale, can be efficiently studied in the RIDME experiment (see section IV).

Next, we turn to modeling of the RIDME data. As in other PDS experiments, the RIDME time signal consists of a decaying electron intermolecular “background” from distant electrons in the EPR sample and an intramolecular “form factor”. These two contributions are factorizable.^21^ As explained above, the decay in RIDME traces is additionally strongly affected by the electron–nuclear couplings—it is much steeper in a protonated environment than in a deuterated environment.^21,24^ This is in contrast to DEER, where the shape of the signal decay is determined only by the intermolecular electron–electron couplings and a change of the phase memory time *T*_m_ has only a global scaling effect on the DEER trace (if we disregard filtration effects in heterogeneous systems^32,41^). In the past few years, the main features of the RIDME background have been investigated and understood, which covers a theoretical description and suitable fit functions.^21,24,42^ Furthermore, RIDME time traces are affected by a stronger electron spin echo envelope modulation (ESEEM) signal than DEER. To reduce the ESEEM contribution, it is common to conduct RIDME measurements at high magnetic fields and high microwave frequencies. To date, typical microwave frequencies for RIDME experiments are 34–36 GHz (Q band) and 94–95 GHz (W band). The ESEEM contribution can be optionally further reduced by delay time averaging protocols and trace division.^14,22,43^ A RIDME trace typically contains some echo-crossing artifacts that need to be considered in the data processing protocols. These artifacts were systematically studied.^23,44^ Since the partner electron spin flips are relaxation-driven, they can be accelerated or nearly frozen by adjusting the measurement temperature.^14,15,20,45^ This feature is used for instance to separate the electron–electron and electron–nuclear contributions in the RIDME background decay.^24,46^ As a rule, the described measures result in sufficient RIDME data quality for quantitative analysis.

## I Longitudinal Spin-Flip Dynamics in the RIDME Experiment

RIDME experiments with high-spin paramagnetic centers, most importantly Gd(III) and Mn(II), shed some light on the longitudinal spin dynamics in coupled electron spin pairs.^16,17,20,45^ The electron–electron dipolar modulation appears in the RIDME trace provided that the B-spin has changed its spin state during the mixing block. The experimental data reveal only a relative change in magnetic quantum number |Δ*m*_*S*_|, which is an integer between 0 and 2*S*_B_. For a high-spin center acting as a B-spin in the RIDME experiment, the RIDME trace is a linear combination of dipolar evolution signals corresponding to the fundamental dipolar frequency and those for which dipolar frequency is stretched by a factor 2, 3, etc., up to the maximum possible factor of 2*S*_B_. Thus, one speaks of “dipolar frequency overtones” or “dipolar frequency harmonics”, and the corresponding statistical weights are called “overtone coefficients”. In a high-field and high-temperature approximation, limiting probabilities for all starting state/final state combinations of B-spin are equal. Assuming that the spin flip is a Markov process, the expected limiting distribution of overtone coefficients is obtained by counting all (2*S*_B_ + 1) × (2*S*_B_ + 1) transitions with equal weights and sorting them by the |Δ*m*_*S*_| values. We refer to this pattern as thermodynamic equilibrium. The key experimental finding is that in the high-spin RIDME data the spin-flip ratios are stationary but do not correspond to thermodynamic equilibrium, even at the longest mixing times. In the case of eight-level Gd(III) centers (electron spin *S*_B_ = 7/2), the overtones with |Δ*m*_*S*_| = 0, 1, 2, 3, 4, 5, 6, and 7 are expected with the ratios 4:7:6:5:4:3:2:1. For a Mn(II) spin center with *S*_B_ = 5/2, the equilibrium ratio series is 3:5:4:3:2:1 (|Δ*m*_*S*_| = 0, 1, 2, 3, 4, and 5). RIDME experiments on Gd(III)–Gd(III) and Mn(II)–Mn(II) spin pairs revealed immediately that such statistics are not applicable.^16,20,45^ In both cases, only the magnetic dipolar contributions with |Δ*m*_*S*_| = 0, 1, 2, and 3 were observed, with a different stationary pattern for Gd(III) and Mn(II). Higher-|Δ*m*_*S*_| contributions did not have statistically important weights. At short spin–spin distances (<3 nm), only the contributions |Δ*m*_*S*_| = 0 and 1 remain significant, and the contribution of |Δ*m*_*S*_| = 1 gradually decreases with decreasing spin–spin distance. The latter effect indicates the influence of magnetic dipolar coupling between paramagnetic metal centers on the spin flip-flop probabilities below a certain distance threshold. This was supported by showing that the overtone suppression due to the finite excitation bandwidth of the microwave pulses only does not reproduce the experimental data.^47^

**Figure 3:**
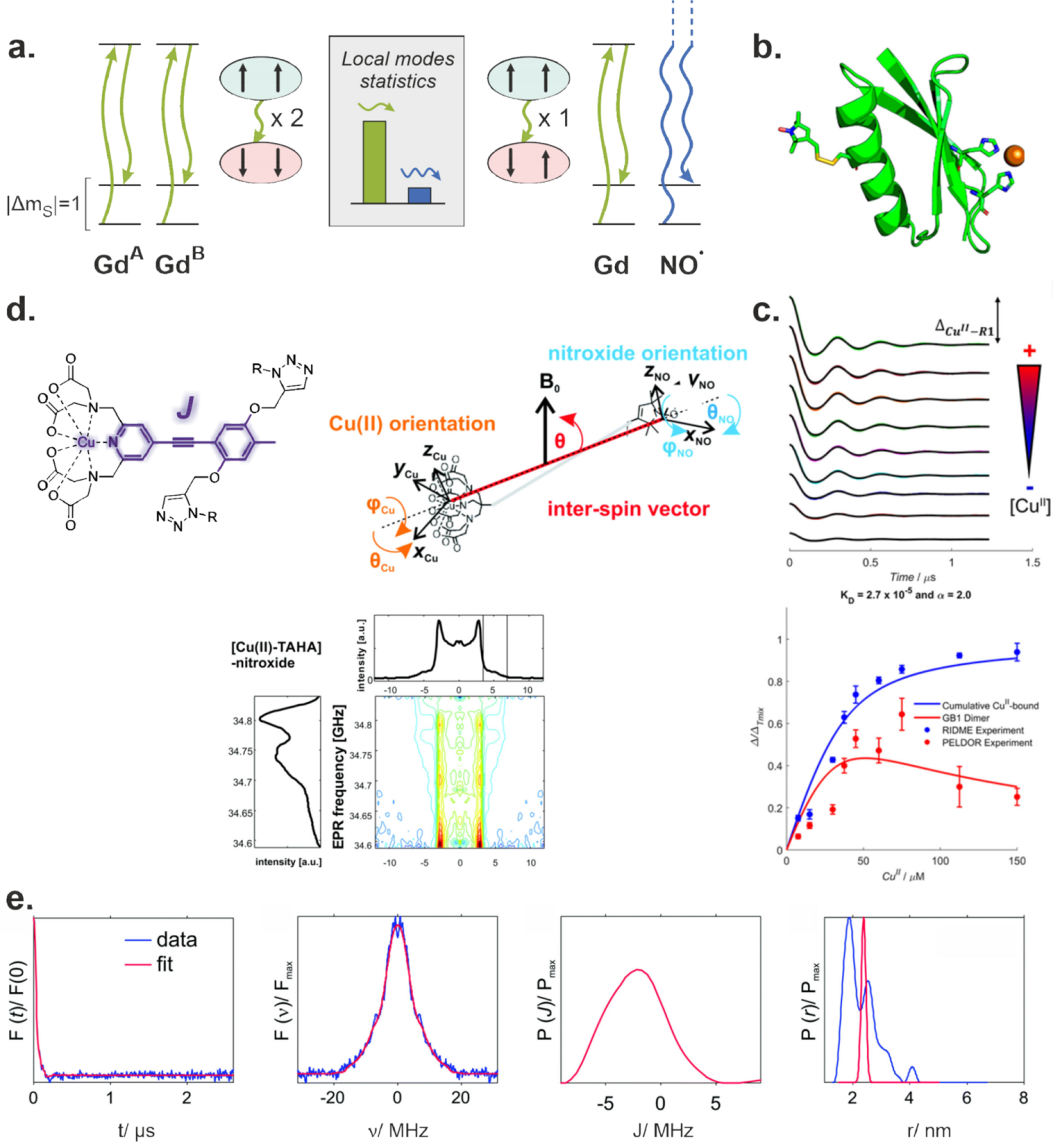
Overview of RIDME applications in EPR studies of metal centers: (a) Low-frequency local matrix vibration modes (green arrows) cause different relaxation kinetics in Gd(III)–Gd(III) biradicals (two identical paramagnetic species) compared to the Gd(III)–nitroxide case (nonidentical paramagnetic species). Low-frequency modes (green arrows) are more populated than the high-frequency modes (blue arrows) and can flip both Gd(III) spins in Raman relaxation events. (b) Nitroxide-labeled GB1 protein with Cu(II)-binding center.^27^ (c) (top) Buildup of RIDME modulations in dependence on the Cu(II) concentration and (bottom) dependence of RIDME and DEER modulation depths on Cu(II) concentration in the GB1 protein dimerization experiment.^27^ (d) (top left) Chemical structure of Cu(II)–PyMTA center conjugated with an aromatic linker—an example of a building block for metal-based biradicals with Heisenberg exchange coupling through chemical bonds. (top right) Polar coordinates defining orientation averaging in a Cu(II)–nitroxide biradical. (bottom) 2D RIDME plot correlating nitroxide’s EPR frequency and dipolar frequency in a rigid Cu(II)–nitroxide biradical (Cu(II) orientations were averaged by the RIDME mixing block).^23^ (e) Extraction of a distribution of exchange couplings in a Cu(II)–nitroxide biradical performed from DEER data. From left to right: form factor RIDME trace in the time domain (blue) and its best fit (red); RIDME form factor (blue) and its best fit (red) in the frequency domain; and corresponding distributions of the exchange coupling: true (red) distance distribution, used for computing exchange couplings, and apparent (blue) distance distribution computed without taking exchange interactions into account.^29^ The plots in (b) and (c) were adapted from ref (27). CC BY 4.0. The plots in (d) were adapted from ref (23). CC BY-NC 3.0. The plots in (e) were adapted from ref (29). CC BY-NC 3.0.

The stationary pattern of dipolar overtones at longer spin–spin distances cannot be explained by the intramolecular dipolar coupling. There are some discussions on the possible mechanisms of such a stationary balance with almost no dependence on interspin distance, temperature, and mixing times.^20,47^ The most realistic phenomenological model published so far and capable of reproducing the nearly constant overtone pattern assumes the presence of both single-quantum and double-quantum flips in the partner spin’s relaxation kinetics.^20^ One quite realistic hypothesis that, to the best of our knowledge, has not yet been discussed in the literature suggests the resonance character of the spin interaction with the local vibrational modes (Figure 1I). Consider a pair of alike spin centers and an excited local mode vibration that is most efficiently coupled with one of the two spins. The local vibrational modes involved in electron spin flips are usually in the low-frequency region of the vibrational spectrum, and therefore, they are delocalized in a volume exceeding the size of the molecule. Thus, the same mode would also be strongly coupled to the other spin in the spin pair (Figure 3a). This effect should be particularly strong at the narrowest central transition of the Kramers-type Gd(III) or Mn(II) ions, where both spins in the spin pair have close resonances. In such a case, the frequency difference of the two local vibrational modes most commonly involved in either the Orbach or Raman relaxation process can be resonant with both spins simultaneously. This means that once there is a high population of vibrational quanta in a particular local mode that is efficiently coupled to the spins, the spin-flip probability increases for both spins in the spin pair. In the RIDME experiment, detection is typically performed exactly on this central transition. Hence, to a rough approximation, one can assume that the flips of the partner spin between the ±1/2 states induce the decay of the detected spins due to the strongly correlated relaxation of both the detected spin and the partner spin. Consequently, the detected signal is formed when the spin flips of the partner spin happen within the subsets of the levels [−7/2, −5/2, −3/2, −1/2] and [1/2, 3/2, 5/2, 7/2]. In this oversimplified case, if we repeat the above-mentioned computation of the dipolar overtone statistical weights, we obtain the ratio 4:3:2 for the transitions with |Δ*m*_*S*_| = 1, 2, 3. Higher-|Δ*m*_*S*_| transitions are suppressed in this model since the B-spin must pass through the ±1/2 transition to realize them. Given the roughness of this approximation, the obtained weight ratio of 4:3:2 is not too far from the experimentally extracted values, which are in the range between 5:4:1 and 4:4:2.^20,47^ This hypothesis can conveniently explain the near independence of the dipolar overtone coefficients in the spin–spin distance. Indeed, the characteristic size of a local mode vibration would be at a much larger length scale than the few-nanometer spin–spin distances relevant for PDS. Also, at different temperatures, the population of a particular local vibrational mode would change, while the coupling of this mode to the electron spins would remain practically the same. This is consistent with the experimentally observed temperature stability of the dipolar overtone coefficients in RIDME.^17^ It is so far unknown how such interactions with local modes change at the temperatures where the average numbers of quanta excited for particular local modes decrease and the direct relaxation processes involving a single vibrational quantum start to dominate over the Raman and Orbach processes, which include two phonons in crystals or two local vibrational mode quanta in amorphous solids. One may anticipate, for instance, some substantial changes in the dipolar overtone coefficients in the temperature range where one relaxation mechanism of the high-spin center is overtaken by another one. In a heterospin system, shown as a second example in Figure 3a, the correlated relaxation does not take place, and higher-order transitions should be observed with higher statistical weights, as indeed is reported, e.g., in the Mn(II)–nitroxide RIDME experiments.^48^

For the low-spin paramagnetic centers (*S* = 1/2), such a resonant-local-mode hypothesis can explain why the limiting modulation depth λ_∞_ is sometimes found to be less than 0.5, e.g., in the Cu(II)–Cu(II) and nitroxide–Cu(II) RIDME experiments.^23,26^ Regardless of a particular underlying mechanism, which is still to investigate, the steady-state pattern of dipolar overtone coefficients of the RIDME modulation buildup is a well-established experimental fact: the measured flip statistics of the B-spins do not correspond to the thermodynamic equilibrium but are biased. Consideration of this feature can become necessary in the high-precision studies that quantitatively rely on the modulation depth determined by RIDME (e.g., the metal ion affinity measurements,^25−27,49^ discussed in detail in the next section). In an optimal scenario, the affinity measurements need to be done in a broad range of metal ion concentrations reaching far into the saturation plateau, and the RIDME traces at each concentration need to be measured with long enough mixing time, exceeding 3 times the typical longitudinal relaxation time of the metal ion paramagnetic centers under investigation. The mentioned limiting modulation depth λ_∞_ corresponds to a saturated solution where all binding sites are occupied. With these fulfilled conditions, as was also done in the cited references, one can safely use the modulation depth at a given metal concentration normalized by the limiting value λ_∞_ as a measure of the metal binding probability even if λ_∞_ deviates from its theoretical value. This strategy makes the results unaffected by the specific value of the maximum modulation depth, which can be differently biased due to the complex spin–lattice dynamics.

Under suboptimal conditions, such as low-affinity metal binding sites or proton-rich samples with short decoherence time, it might be difficult to achieve the regimes of mixing times or metal ion concentrations described in the previous paragraph. Consequently, the determination of the saturated value of the RIDME modulation depth may become numerically unstable. The affinity measurements with the RIDME technique might still be feasible with these limitations. We suggest that careful analysis of the spin-flip dynamics of the metal spin centers can help make the parameter λ_∞_ in RIDME experiments more accurately predictable. Having such an additional constraint for affinity curve fitting should also stabilize the determination of the affinity constant.

The resonant flips of the detected and relaxing spins can affect, besides the limiting modulation depth, the apparent modulation buildup time for the given relaxing spin, so that a difference between the RIDME buildup time and the intrinsic longitudinal relaxation time of the partner spins might appear. This phenomenon has not yet been investigated, and besides the overall calibration of the RIDME technique for high-spin paramagnetic centers, such spin-flip dynamics is also directly relevant to the field of quantum computing, with different coupled spin systems being tested as qubits.^50^ In that field, any phenomena related to the spontaneous spin flips, such as also the correlated neighbor spin flips, are of particular importance, as such events cause spontaneous information losses. Here, RIDME is one of the efficient spectroscopic tools to deepen our knowledge of the coupled spin-flip dynamics. Thus, using RIDME in connection with the relaxation properties of qubits or any other spin-coupled system appears to be an attractive new opportunity.

## II Affinity Determination for the Metal Binding Sites in Proteins

Although we propose some developments in this section, these are only aimed at clarifying the applicability range, while the affinity determination using RIDME can already be considered a ready-to-use technique. RIDME can be formally attributed to a family of pump–probe techniques, alongside DEER. However, in the case of RIDME we probe a fraction of electron spin centers in the sample by applying microwave pulses, whereas the “pump” events take place spontaneously due to the partner spin flips (longitudinal relaxation). Such “pump” flips therefore take place at all spectral positions and do not depend on the spectrometer bandwidth limitations. Thus, for paramagnetic centers with broad EPR spectra, it is easier to record RIDME data with large modulation depth, while achieving high modulation depth in DEER experiments, where one pumps spins also with microwave pulses, is often a demanding task.

Due to its modulation depth advantage, stemming from the mentioned virtually infinite-bandwidth pumped-spin excitation, RIDME can be quite efficiently used to determine metal ion binding equilibria (Figure 1, II).^25−28,49^ The key value exploited in this approach is the modulation depth, which is more related to the relaxation properties of the paramagnetic centers than to the specific type of interaction that is responsible for the modulation. For instance, modulation depth can be used in the same manner for the case of pure dipolar interaction between two spins and for a mixed dipolar and exchange interaction case. The RIDME-based approach to determine metal binding equilibria was first tested on the specially designed double-histidine Cu(II) binding motifs in GB1 proteins (Figure 3b,c).^25,26,49^ The concept of binding polynomials, originally developed for isothermal titration calorimetry,^51^ was adapted for the RIDME data analysis.^49^ The technique was later also applied to follow the metal-templated dimerization of GB1.^27^ Changes in relaxation behavior upon metal ion binding and its effect on the RIDME modulation depth determination have been reported.^52^ The possible differences of the EPR spectra and the relaxation properties of the metal ions in free and bound forms would affect the determination of binding constants with the RIDME-based approach due to the differences in the EPR spectral densities at the detection frequency. However, as discussed above, this would be an issue only if the modulation depth cannot be renormalized by its limiting value. A comparison between modulation buildup in RIDME and DEER is one of the possible tests to verify that the binding equilibrium of metal ions is not strongly affected by the stationary character of the RIDME modulation buildup that was discussed in section I. Affinity measurements on different model systems based on both DEER and RIDME have been reported,^27,53^ which makes such comparison clearly feasible.

Note further that as an alternative to the modulation depth determination in RIDME, metal–radical interactions can also be detected via, e.g., the longitudinal relaxation enhancement technique, for which the effective depth parameter describes the true thermodynamic equilibrium rather than a stationary equilibrium (here, the effect depth can be determined as the fraction of radicals with enhanced relaxation).^54,55^ On the one hand, the steep distance dependence of the relaxation enhancement effect (∝*R*^–6^), and accordingly, a relatively narrow sensitivity range, are disadvantages of the relaxation enhancement technique. On the other hand, its possible advantages are that it reports the true equilibrium distribution of bound/nonbound states and that it might potentially work also at ambient temperature,^56^ i.e., in solution without the need to correct for the binding affinity offset due to the shock freezing. These points should be considered in future cross-validation of RIDME-based and relaxation-enhancement-based approaches to affinity determination in order to evaluate the possible effect of freezing and the transient nature of the modulation depth in RIDME.

During freezing, the sample temperature decreases over a finite amount of time. Thus, before immobilization in a glassy matrix, metal ions may dynamically shift their binding equilibria following the gradually changing local temperature. Hence, there is an interplay between the characteristic time of re-establishing the metal ion binding equilibrium and the characteristic freezing time, which can be different for different freezing protocols. If the two time scales are comparable, then depending on the characteristic freezing time, the binding equilibrium might shift in one or another direction. There is a worked example demonstrating that exothermic binding tightens upon freezing and endothermic binding does the opposite.^26^

## III Orientation-Selection-Free Detection and Quantification of Exchange Coupling Distributions

In this section, we discuss an already well-developed approach that can be directly exploited to determine exchange coupling distributions in rigid biradicals. Due to the same advantage of the nearly infinite bandwidth of the pumped spin inversion, RIDME is well-suited for detecting entire distributions of exchange couplings in paramagnetic-metal-based spin pairs (Figure 1, III). Exchange-coupled spin pairs are of substantial interest for single-molecule-based spintronics, molecular electronics, and photovoltaics.^57−60^ Also, exchange-coupled systems appear in the studies of artificial photosynthetic systems.^61,62^ The specific strength of EPR spectroscopy is in the ability to determine weak exchange couplings with high sensitivity, down to subnanomolar amounts of spins.^63^ So far, quite often, continuous-wave (CW) EPR spectroscopy in solution is exploited to determine a single value of the exchange coupling averaged over the molecular motion.^64−66^ Also, in the frozen or solid state, CW EPR is regularly applied to determine an average exchange coupling value.^63,67^ However, a very important and to date insufficiently studied characteristic of such exchange-coupled systems is the variability of the exchange coupling upon molecular structure distortions. Even small torques or strains can significantly affect the shape of singly occupied molecular orbitals and the strength of the exchange coupling while leaving the spin–spin distance and thus the through-space magnetic dipolar coupling between the two spins unaffected. As a result, the appropriate characterization of exchange-coupled systems needs to include the determination of the distribution of exchange couplings, which can only be achieved in EPR using PDS experiments.^29,68^

An important issue of obtaining the correct distribution of exchange couplings is the need for angular averaging of the mixed exchange-dipolar coupling pattern. This is particularly difficult in magnetically coupled molecular systems with paramagnetic metal ions, as they are characterized by broad EPR spectra, which can be beyond 100 mT in width.

In the first publication that discussed the need to take into account the distribution of the exchange couplings, X-band Cu(II)–nitroxide DEER was exploited.^69^ The orientation selection was also analyzed on a model compound with a very similar structure but a somewhat different spin–spin distance.^70^ However, at that time, the DEER measurements to record the dipolar and exchange coupling data were performed without orientation averaging. Realistically, such averaging also would not be feasible on that compound using the available X-band DEER setup: the Cu(II) EPR spectrum was too broad for the available resonator bandwidth, and also, the Cu(II) and nitroxide EPR spectra overlapped, thus excluding part of the orientation pairs from the averaging protocol (in DEER, pulse and probe positions must be different). As a result, by using what at that time were state-of-the-art measurement and analysis approaches, the authors could obtain only moderate agreement between DEER data and simulations. Thus, the problem of accurate determination of the exchange coupling distribution remained recognized but unsolved for some time.

In this respect, RIDME has a significant advantage over DEER, as it offers the angular averaging over the pumped spins essentially “for free” simply due to the absence of the bandwidth limitations in the RIDME block-relaxation-based excitation (Figure 3d).^23,29,71^ This makes the two-spin angular averaging protocols in RIDME substantially simpler than those in DEER.

The main drawback of PDS, and in particular RIDME, is the rather narrow range of exchange couplings that can be determined (roughly, between 1 and 100 MHz, Figure 3e). This limitation can be mitigated in a two-step experiment design, which can be established, for instance, on exchange-coupled systems with conjugated spacers of variable length between paramagnetic centers. In the first step, the variability of the exchange interaction upon molecular strains can be determined for an appropriate (longer) spacer so that the exchange couplings are distributed over the most sensitive PDS range. In the second step, the scaling of the mean coupling with the spacer length is independently determined, e.g., from CW EPR or magnetometry. Such an approach appears to offer some unique capabilities, and we anticipate that it will conceive in the future another “nonconventional” application field for RIDME spectroscopy.

## IV Quantitative Longitudinal Spectral Diffusion Analysis of RIDME Measurements

In the following three sections, we discuss a very new concept of using the RIDME technique, which we have been developing only over the last 3–4 years. Despite this short time, it has already turned into a rich playground for new developments. Furthermore, within this new concept, quite a few ready-to-use solutions to characterize sample heterogeneity and local structure have already been developed and are available for applications.

The shapes of time traces in RIDME experiments are affected by not only electron–electron dipolar interactions but also by the electron spin interactions with the surrounding magnetic nuclei. While being a disadvantage for the electron spin–spin distance measurements, this feature of RIDME traces leads to new interesting opportunities relating RIDME data to some aspects that are important for paramagnetic NMR and hyperpolarization.

In DEER, the pump pulse frequency is set away from the excitation bandwidth of the detection pulses. Hence, the evolution of the ensemble of detected spins is essentially the same at all positions of the pump pulse, disregarding the electron dipolar coupling. In RIDME, the moving mixing block consists of two pulses of the same frequency as the detection part of the pulse experiment. As a result, the RIDME time trace contains a decaying background contribution because the electron spin transverse evolution due to interaction with the nuclear bath is interrupted by the mixing block at different moments. Here it is essential to include the homonuclear coupling into consideration because otherwise the hyperfine interaction is perfectly refocused.^24,72−74^ The nuclear spin dynamics during the mixing block affects the longitudinal grid, which is formed after the transverse magnetization of the excited ensemble of electron spins is directed along the magnetic field by the first π/2 pulse of the mixing block. This nonequilibrium part of the electron spin longitudinal magnetization undergoes longitudinal spectral diffusion (LSD) due to the spin diffusion in the surrounding proton spin bath (more specifically, only the protons beyond the spin diffusion barrier).

**Figure 4:**
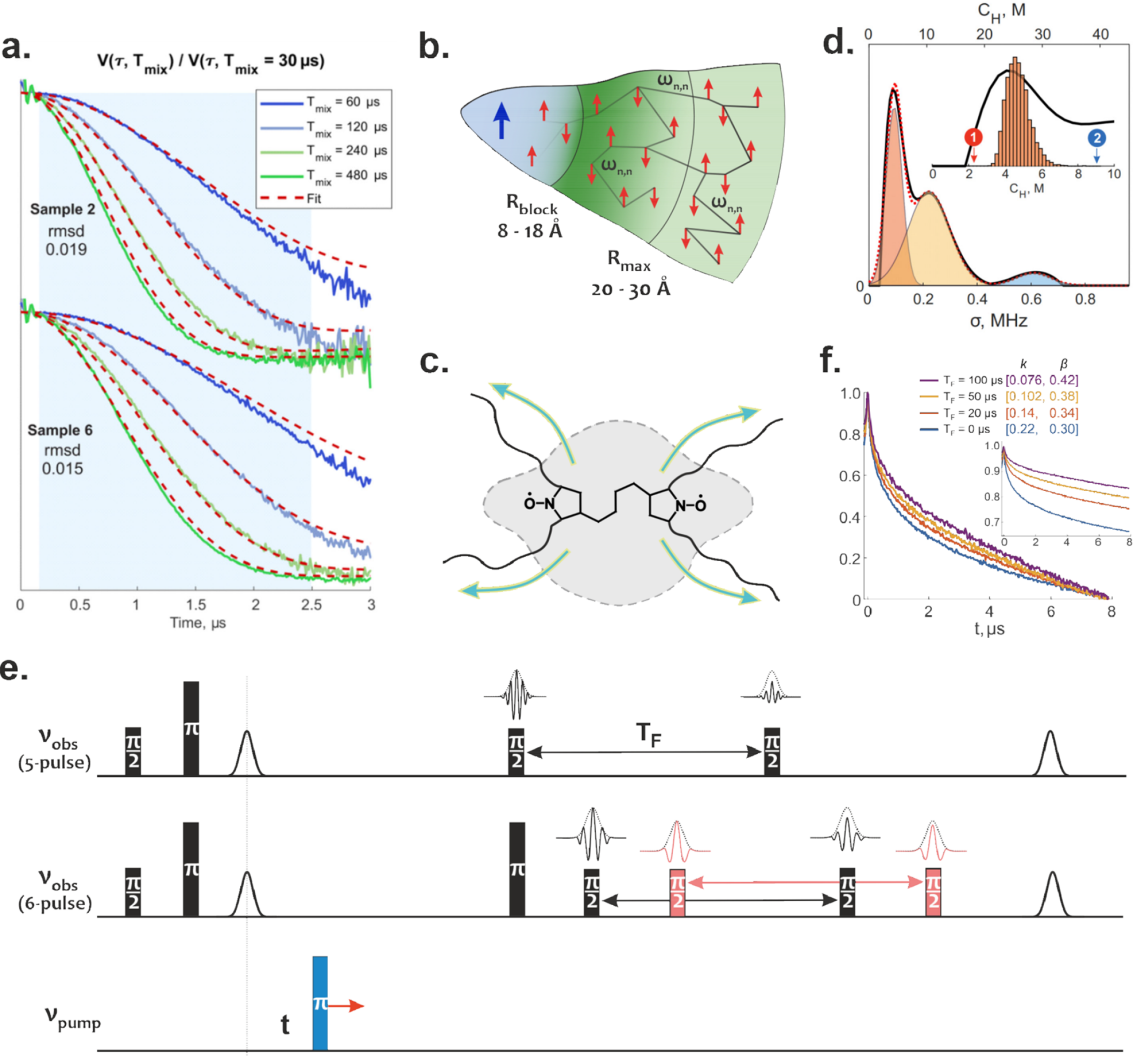
(a) Global fit of the series of ih-RIDME traces after the division by the reference trace with the lowest mixing time. Adapted from ref (24). CC BY-NC 3.0. (b) Formal separation of the nuclear spin bath into a frozen core (gray), too-distant spins (light green), and the middle part where the difference of the hyperfine couplings is balanced by the internuclear couplings. The black lines connect close nuclear spins with the opposite nuclear states which, therefore, may undergo flip-flop transitions and participate in the nuclear spin diffusion. (c) Schematic representation of a biradical with large flanking groups around paramagnetic moieties—a typical polarization agent in DNP. The quasi-elliptical area in the background indicates the presence of the spin diffusion barrier in the vicinity of the biradical. (d) Distribution of local proton densities in spin-labeled barley β-glucan (BBG) obtained via analysis of ih-RIDME traces.^33^ The inset shows a comparison between the ih-RIDME-based proton concentration distribution (black line) and the same distribution calculated by Monte Carlo modeling of BBG conformational distribution (histogram). (e) Pulse scheme of five- and six-pulse hyperfine-filtered DEER experiments (hf-DEER) derived based on the four-pulse DEER experiment by adding the longitudinal block. Spectral diffusion processes during this block filter out the EPR signal of proton-rich spins sites. Microwave pulses are labeled by their flip angles. *T*_F_ stands for “filtering time”. In the six-pulse version, the frequency of the polarization grating and therefore the filtering effect can be flexibly tuned independently on the chosen dipolar evolution time. (f) Shape evolution of the dipolar trace of BBG in the five-pulse hf-DEER experiment with increasing length of the mixing block featuring the correlation between the interlabel distances and local proton densities.^33^ The plots in (d) and (f) were adapted from ref (33). CC BY 4.0.

We have recently developed a mathematical model and a corresponding numerical method to quantitatively describe this effect, fit the intermolecular hyperfine RIDME (ih-RIDME) data, and obtain the characteristic parameters of the homonuclear spin bath around the paramagnetic species.^24^ The only two fit parameters of this model are the width of the electron spin resonance shift distribution, σ, from hyperfine couplings and the reduced spectral diffusion coefficient, *D*/σ^3^. The former parameter (σ) describes the local nuclear spin concentration (in the homogeneous nuclear spin distribution case). In this context, we correct a numeric mistake in eq 4 in ref (24) (the constant *B* is 0.222 Mrad/s nm^3^ instead of 0.0555 Mrad/s nm^3^ as given in the cited reference). The latter parameter (*D*/σ^3^) appears to be independent of the nuclear spin concentration and is thus a characteristic property of a particular frozen amorphous matrix type. The RIDME decay traces reveal a precise linear scaling with respect to the bulk nuclear spin concentration. In a broad range of mixing times, the RIDME traces can be fitted globally with the two fit parameters introduced above with good accuracy, as exemplified in Figure 4a. Importantly, the linear scaling with the proton concentration is more sensitive than approximately the square root of concentration scaling for the two-pulse Hahn echo experiment (Figure 1, IV).^24,75^ Due to the global fit of several RIDME traces with different mixing times, the fit results demonstrate good stability and rather narrow uncertainties of σ and *D*/σ^3^. The developed ih-RIDME-based approach appears to be sensitive enough to discriminate between different types of protonated molecules in the frozen glassy matrix around electron spins. In particular, the ih-RIDME datasets for protonated water in deuterated glycerol or protonated glycerol in deuterated water in the frozen water/glycerol glasses were sufficiently different to result in clearly separated sets of fit parameter pairs obtained from the ih-RIDME data.^24^

The proton-flip-flop-related contribution to electron LSD is temperature-independent, while some other contributions, e.g., nuclear longitudinal relaxation, are anticipated to be temperature-dependent in the electron LSD. With ih-RIDME-based analysis, we thus obtain a tool to investigate the specific dominant relaxation mechanisms of the nuclear spins in the vicinity of a paramagnetic center. This can potentially evolve into a rather broad variety of ih-RIDME applications in the studies of soft matter, DNP methodology, and paramagnetic relaxation enhancement (PRE) calibration studies. In particular, the ih-RIDME technique might appear efficient for investigating the secondary polarization transfer pathways related to the proton spin diffusion after primary polarization transfer from an electron spin to a nuclear spin. This is currently a relevant topic under discussion in the DNP community.^31^ Such a technique might also find applications related to characterization of magnetic resonance imaging (MRI) agents, as MRI is also directly related to the actual distributions of electron–nuclear couplings.^76^ Note also that besides the information on the width of the distribution of electron spin resonance frequency offsets due to the electron–nuclear couplings, fitting of ih-RIDME data provides the spectral diffusion coefficient *D* and its reduced analogue *D*/σ^3^, which is concentration-independent and can be used as a universal measure of the characteristic “proton connectivity” in a particular type of glassy material. The value of *D*/σ^3^ is already averaged over a large number of protons around the electron spin and is therefore a good measure of, e.g., the polarization transfer rate through the bulk of a proton-containing glass. The ih-RIDME technique revealed quite a good sensitivity to the model fit parameters,^24^ thus offering a method to sort different glassy matrices in terms of their proton bath connectivity.

Another prominent and important relation to the DNP study appears from how the local proton bath is structurally seen in ih-RIDME (Figure 4b). Very distant protons (>3 nm) are characterized by weak interactions with the electron. Hence, their spin state change contributes insignificantly to the signal dephasing and can be neglected. Alongside with this, the protons in proximity (<0.5–0.6 nm) are strongly coupled to the electron spin, and homonuclear spin–spin coupling cannot efficiently mix the nuclear levels and stimulate a flip-flop transition.^72^ Thereby, the nuclear spin reservoir is separated into a “frozen” core and a “dynamic area” beyond the border of that core region. This formal border between the two regions, known as a spin-diffusion barrier, is an important object in DNP studies.^77−79^ In this context, we make two notes. First, the diffusion barrier is not a sharp surface but rather a zone of a gradual change in the relation between the electron–nuclear and nuclear–nuclear interaction strength. Second, the position of the spin-diffusion barrier is not fixed at a particular distance from the electron spin but rather depends on the angle between the static magnetic field and the electron spin–nuclear spin vector direction, on the coupling of the nuclear spin to its nearest neighbor nuclear spin, and on the orientation of this nuclear spin–spin vector.^78^ Consequently, the spin diffusion barrier positions might be substantially distributed in space for different types of nuclear spin pairs. Such effects are, for instance, important for polarization transfer through macromolecules characterized by an ensemble of substantially different conformations, thus making ih-RIDME a method to study such systems. In addition, the shape of the diffusion barrier in the biradicals (Figure 4c) is not a result of the direct embedding of the barrier shapes corresponding to individual electrons. This is because local gradients of the electrons’ magnetic fields can both add up and compensate for each other, thereby interfering. This motivates one to study spectral diffusion in biradicals for the purposes of cross-effect DNP, where again ih-RIDME is a good method to apply.

In our view, ih-RIDME is a promising tool for quantitative investigation of the diffusion barrier and related phenomena such as spin diffusion kinetics in the vicinity of the paramagnetic centers (Figure 4c). It has been already proven that the distribution of blocked and nonblocked proton spin pairs in the water/glycerol glasses is strongly dependent on the bulk proton concentration: the effective cutoff blocking radius for the proton spin diffusion scales as the inverse cube root of the bulk proton concentration.^24^ By using ih-RIDME data and appropriate modeling, it should be possible to go beyond the single cutoff radius value as an approximation for the proton spin diffusion blockage in the vicinity of paramagnetic centers. Making detailed analysis of RIDME data on such samples, including molecular modeling for the proton positions distributions and accurate quantum-mechanical description of the nuclear spin pair dynamics for the spin diffusion, appears to be an efficient new approach to elucidate details of the nuclear polarization transfer in the vicinity of paramagnetic centers, e.g., in relation to the DNP experiments.

From this perspective, one may consider the ih-RIDME technique to be a complement to the ESEEM and electron–nuclear double resonance (ENDOR) techniques, which mainly target the nuclear spins within the blocked spin diffusion region.

## V Quantification of Heterogeneous Local Proton Spin Densities with ih-RIDME

The ih-RIDME technique is not the only EPR method to determine electron–nuclear interactions. Different techniques based on CW EPR progressive power saturation,^80^ transverse electron spin relaxation,^72,73^ ESEEM,^65,81^ or Overhauser DNP^82−84^ were shown to reveal the deuteron or proton accessibility to the paramagnetic centers. From the listed methods, except for the transverse relaxation measurements, the electron–proton interactions could be characterized only by the mean value. For the transverse electron spin echo decay, so far only rather computationally expensive methods for calculation of the time traces based on an atomistic model of the proton distribution have been reported.^72,73^ Such calculations show a rather good match with experimental data, but this approach is quite difficult to incorporate into a fitting procedure with varying local proton surrounding of the electron spins.

The ih-RIDME technique appears to be richer in information than the previously developed methods. Importantly, in the ih-RIDME data fitting, the specific values for the fit parameters have a direct physical interpretation. Due to these features of the new technique, ih-RIDME can be transformed into a tool to quantify the local heterogeneity in the samples characterized by a distribution of local proton concentrations around paramagnetic centers. A proof-of-principle example of such measurements and the local heterogeneity quantification has been just recently reported by the authors.^33^ In the cited work, soluble dietary fibers (DFs) of barley β-glucan (BBG) were investigated. Such DFs are prone to weak interchain interactions and form in solution reversible condensates of a variable number of chains. It was demonstrated by the DEER experiments with variable transverse evolution times that when BBG DFs interact with small molecules, the chain contact statistics are affected.^32^ It was later also demonstrated by DEER, RIDME, and molecular modeling that the main contribution to the local chain contacts is due to the interactions between different chains in the aggregates, not by the intrachain contacts.^37^ Thus, if the solvent is deuterated, which is needed to create a proton contrast, then the spin labels in BBG DF samples can probe the heterogeneity of local sugar monomer densities arising from conformational flexibility and aggregation. This can be correspondingly translated to a distribution of the local proton concentration values. This heterogeneity depends on the DF concentration and on the interactions with small molecules and thus appears to be a good test system for the ih-RIDME technique.

The ih-RIDME analysis of spin-labeled BBG DFs in free form as well as in interaction with small dye molecules and divalent Mg(II) ions resulted in distributions of the σ values, showing a characteristic three-peak pattern with bell-like peaks corresponding to the free single BBG DF chains, “weak” aggregates, and more dense “strong” aggregates (Figure 4d). The free DF chains peak was compared to the results of molecular modeling (which was also validated by comparison to DEER-based distance distributions), and a good match was found, thus confirming that the distributions determined from the ih-RIDME proton concentration are close to the reality.

Note that the “contacts” as seen by ih-RIDME are not necessarily related to strong binding with defined coordination geometry. Such binding can be rather well determined, e.g., by NMR as the presence of characteristic peaks with perturbed chemical shift compared to the free chain NMR peak positions. In ih-RIDME, one also observes weak interchain contacts that might lead to no defined NMR peak shifts as well as just near approach situations when two chains are not in direct contact but are within the approximately 3 nm vicinity of each other. This is an interesting new type of information, and developing structural tools based on the ih-RIDME technique is thus an attractive new challenge for EPR spectroscopists.

We emphasize that ih-RIDME is the first example in EPR spectroscopy where the electron spin interactions with the proton bath are described not by a single value but as a 1D distribution of local parameters with corresponding statistical weights. Such a detailed picture might very well serve in the future as a basis for a number of interesting new applications in various research areas ranging from soft materials research to structural biology—where local heterogeneity of the samples is of importance. In particular, in structural biology, the determination of conformational ensembles of partially or fully unfolded biomacromolecules is an interesting and challenging task, addressable by only a limited number of techniques, such as small-angle scattering (SAS), FRET, and PDS. Here, ih-RIDME might substantially contribute by providing chain contact statistics and become one of the standard characterization techniques.

Also, ih-RIDME data might be used to stabilize fitting procedures for the SAS technique on dispersed solutions of partially or fully disordered proteins or other biomacromolecules. The problem of reconstructing the shapes of scattering macromolecules from SAS data is ill-posed. Unless this is done with all-atoms molecular modeling, a typical approach to perform such shape reconstruction consists of randomly combining smaller bids of a given scattering density into a larger shape.^38,85^ For folded biomacromolecules or biomolecular complexes, the scattering density of such elementary bids can be rather well predicted a priori, as the density in the folded domains of, e.g., proteins is reasonably well-known and does not vary a lot. In the case of analyzing SAS data for fully or partially unfolded biomacromolecules, in the reconstruction of the conformational ensemble one must allow for the local scattering densities to vary significantly, thus creating an additional large set of fitted parameters or a parametric distribution. This further destabilizes the fitting procedure. Accordingly, data from ih-RIDME can be used to convert the local proton densities into the total scattering density distribution, which will then turn into a fixed input rather than additional fit parameters. Such complementary use of the ih-RIDME data for SAS analysis thus appears to be an interesting application, especially taking the popularity of SAS studies.

## VI Perspectives on 2D Correlation Experiments Based on ih-RIDME

The efficient and robust mathematical analysis for the inverse ih-RIDME data processing (calculation of the local proton concentration distribution from a set of RIDME traces) is still a topic for further investigation. The straightforward regularization-free fitting demonstrated rather stable performance of such computations.^33^ As a next step in designing RIDME-based spectroscopic tools, one can attempt to correlate the ih-RIDME output with the filtration-prone electron spin–spin distance distributions from DEER or some other PDS measurement.

In weakly ordered or disordered systems with local heterogeneities, it is quite natural to anticipate that the hyperfine interactions between electron and nuclear spins may correlate with the electron–electron dipolar coupling strengths. Consider a solution of a doubly spin-labeled IDP in a deuterated buffer. Then, in the EPR experiments, all observable protons belong to the protein chain and longer interspin distances correlate with lower local proton densities (extended chain conformations), while shorter interspin distances correlate with higher local proton densities (compact chain conformations). An opposite correlation can be imagined in the case when an unfolded polypeptide chain binds to the surface of a folded protein domain. In this case, the free chain might have on average more compact conformation but lower local proton density, while if the polypeptide binds on the folded protein domain in an extended conformation, this would correspond to longer interspin distances but higher local proton densities due to the protons from the folded domain in the vicinity of spin labels.

Figure 1, VI presents an artificially generated example of two such correlated distributions, which are different but result in the same 1D distributions of electron–electron distances and local proton concentrations. Making a correlation between these two distributions in a 2D experiment is then a valuable opportunity. Here the quality of the corresponding time traces plays an important role (the absence of strong echo crossings or other types of time-domain artifacts is desirable). We have proposed pulse sequences to combine the ih-RIDME features (hyperfine-driven decays with accurately known “analytical” form) and DEER’s robustness for the determination of electron dipolar couplings (Figure 4e).^33^ In these hyperfine-filtered DEER (hf-DEER) experiments, the position of the pump pulse (*t*) is one dimension and the duration of the filtering block (*T*_F_) is the second dimension with the freedom of nonuniform sampling. The just-published example of a BBG DF study (Figure 4f) reports for the first time the overall quality of the time-domain signals of such correlated RIDME–DEER data. For DFs, which are stochastically spin-labeled, the distance distributions need to be computed from molecular modeling of chain conformations by MC or MD. If this approach is applied to the site-specifically doubly labeled protein or nucleic acid molecules, then 2D correlation plots might be potentially computed without involving molecular modeling. This appears to be an interesting experiment especially in relation to studies of IDPs, IDRs, and their interactions with other biomolecules.

## Perspectives to Apply ih-RIDME-Based Methodology in Structural and Molecular Biology Studies

Proton distributions with strong local heterogeneity, analogous to the ones in DFs, are anticipated for biomolecular samples, such as samples undergoing liquid–liquid phase separation (LLPS). Thus, it would be quite interesting to study LLPS with the newly proposed ih-RIDME technique and the 2D RIDME–DEER correlation technique hf-DEER. While the micrometer-scale morphology of liquid droplets formed upon the LLPS in biomolecule solutions are studied by microscopy and different fluorescence-based partitioning techniques,^86,87^ the shorter-length-scale morphology and the conformational distributions of the biomolecules inside liquid droplets are difficult to access. Here, EPR and, more specifically, the PDS methodology is a clear method of choice,^40,88−90^ and also interesting perspectives for the ih-RIDME technique can be anticipated. In particular, it would be tempting to test whether ih-RIDME can also efficiently quantify the statistics of biopolymer contacts in liquid droplets. Such contact statistics would likely include interactions in a quite broad range of affinities: rather stable specific RNA interactions with the RNA recognition motifs, specific binding sites in the folded protein RNA-binding domains, and weak transient interactions between IDR chains, between IDRs and RNAs, and between IDRs and surface sites in the folded protein domains.

An important obstacle to keep in mind for such weakly interacting systems is that solvent deuteration or adding point mutations for SDSL is no longer a sufficiently weak perturbation. The SDSL/EPR methodology on such systems requires careful calibration and evaluation of the offsets. For folded protein domains, in principle, one might be able to define the interaction sites on the protein surface and then place spin labels at positions that do not interfere with the intermolecular interactions. This is a typical way to avoid substantial changes in protein function upon spin labeling. However, for unfolded proteins or protein domains, all residues come into dynamic contact with other biomolecules, and thus, a particular residue mutation and spin labeling has a higher risk of causing a substantial effect, and this risk is more difficult to evaluate. Thus, it is advisible to always check for such effects experimentally (e.g., with functional or LLPS assays) before using the EPR data on certain spin-labeled biopolymer mutants.

It is already known to us from our own ongoing research and from personal communications with researchers working in the field of LLPS that, at least for some IDP systems, the solvent isotope effect (deuteration/protonation of the solvent) appears to be strong enough to lead, e.g., to spontaneous protein precipitation. Such effects indicate a strong dependence of IDP conformations and chain–chain interactions on the residue hydration properties. We believe that even on such systems, the use of solvent deuteration and ih-RIDME-based techniques might eventually become possible and valuable. However, appropriate compensation for the hydration isotope effects has to be first designed in such cases, e.g., based on the use of chaotropes, solvent mixtures, pH adjustment, and possibly some other procedures. Thus, the chain conformations and contacts in some IDP systems might be possible to study only in a tight connection to a detailed analysis of the hydration effects.

In summary, we have briefly discussed some interesting features of the RIDME experiment which may lead to direct practical advantages so that the new spectroscopic tools can be designed and a broader range of applications can be targeted in the near future. One of the main aims of this Perspective was to attract the attention of the scientific community to the availability of such new opportunities and, at the same time, to the need for further investigations in these directions to establish the listed methodologies as robust spectroscopic tools. The currently acutely researched areas we were mentioning throughout the discussions are quite diverse, ranging from understanding some so far obscure aspects of paramagnetic NMR and DNP techniques to research on new materials, quantum computing, or still rather new topics in cellular and molecular biology of investigating membraneless organelles and their relation to function and pathology in protein–RNA systems. We also hope that the present overview will boost studies on how to adapt more pulse EPR methods to these important areas of research.
